# A Methionine-Induced Animal Model of Schizophrenia: Face and Predictive Validity

**DOI:** 10.1093/ijnp/pyv054

**Published:** 2015-05-19

**Authors:** Lien Wang, Amal Alachkar, Nayna Sanathara, James D. Belluzzi, Zhiwei Wang, Olivier Civelli

**Affiliations:** Departments of Pharmacology (Ms L. Wang, Dr Alachkar, Ms Sanathara, Dr Belluzzi, Dr Z. Wang, and Dr Civelli), Pharmaceutical Sciences (Dr Civelli), and Developmental and Cell Biology (Dr Civelli), School of Medicine, University of California, Irvine.CA.

**Keywords:** Schizophrenia, methionine, mouse model, face validity, predictive validity

## Abstract

**Background:**

Modulating the methylation process induces broad biochemical changes, some of which may be involved in schizophrenia. Methylation is in particular central to epigenesis, which is also recognized as a factor in the etiology of schizophrenia. Because methionine administration to patients with schizophrenia has been reported to exacerbate their psychotic symptoms and because mice treated with methionine exhibited social deficits and prepulse inhibition impairment, we investigated whether methionine administration could lead to behavioral changes that reflect schizophrenic symptoms in mice.

**Methods:**

l-Methionine was administered to mice twice a day for 7 days.

**Results:**

We found that this treatment induces behavioral responses that reflect the 3 types of schizophrenia-like symptoms (positive, negative, or cognitive deficits) as monitored in a battery of behavioral assays (locomotion, stereotypy, social interaction, forced swimming, prepulse inhibition, novel object recognition, and inhibitory avoidance). Moreover, these responses were differentially reversed by typical haloperidol and atypical clozapine antipsychotics in ways that parallel their effects in schizophrenics.

**Conclusion:**

We thus propose the l-methionine treatment as an animal model recapitulating several symptoms of schizophrenia. We have established the face and predictive validity for this model. Our model relies on an essential natural amino acid and on an intervention that is relatively simple and time effective and may offer an additional tool for assessing novel antipsychotics.

## Introduction

l-Methionine, an essential amino acid, is required for the generation of *S*-adenosylmethionine (SAM), the primary methyl donor in almost all methylation reactions. Methylation impacts a wide range of substrates, including proteins, phospholipids, polysaccharides, RNA, and DNA (Loenen, 2006). Consequently, methylation reactions affect numerous biological processes and metabolic pathways that are involved in cell proliferation, differentiation, survival, and other cellular functions (Wu et al., 2010; Horvath et al., 2012; Chen et al., 2013; Kim et al., 2014; Roidl and Hacker, 2014). In particular, DNA methylation is an important epigenetic factor that regulates gene expression (for review, see Jurkowska and Jeltsch, 2010; Ehrlich and Lacey, 2013).

A chronic dietary deficit of methionine lowers the concentration of SAM and reduces methylation of DNA cytosine in rats and mice (Wainfan et al., 1989; Niculescu and Zeisel, 2002; Pogribny et al., 2005; Cordero et al., 2013; Takumi et al., 2015). On the other hand, repeated l-methionine administration to mice and rats has been reported to induce hypermethylation of certain genes’ promoters (Tremolizzo et al., 2002; Batra and Verma, 2014; Parrish et al., 2015).

Going back to the sixties and seventies, several studies have reported that methionine administration to patients with schizophrenia exacerbates psychotic symptoms (Pollin et al., 1961; Brune and Himwich, 1962; Antun et al., 1971; Cohen et al., 1974). Changes in methylation were proposed to be the cause of the responses. Indeed, alteration of the methylation pattern has long been associated with schizophrenia. The transmethylation hypothesis of schizophrenia, first described by Osmond and Smythies (1952) in the early fifties of the last century, proposed that increases in the abnormal metabolism of dopamine, noradrenaline, and serotonin, which leads to increases in their methylated metabolites, might be responsible for the psychotic symptoms.

Since then, strong associations between schizophrenia and genes encoding the transmethylation enzymes have been reported in various human populations and experimental animals (Roffman et al., 2008; Lajin et al., 2012; Saradalekshmi et al., 2014). The epigenetic hypothesis of schizophrenia has shown a strong association of abnormal DNA methylation with schizophrenia (van Eijk et al., 2014; Hass et al., 2015; Li et al., 2015). Patients with schizophrenia exhibit global changes in DNA methylation in leukocytes (Shimabukuro et al., 2007) and overexpression of DNA methyl transferase in GABAergic interneurons in the prefrontal cortex (Zhubi et al., 2009).

It has been shown in mice that a prolonged methionine treatment produces behavioral responses that mimic certain aspects of schizophrenia such as social deficit and prepulse inhibition (PPI) (Tremolizzo et al., 2002). An epigenetic mechanism was proposed to account for these behavioral responses. However, this study was not extended to determine whether methionine administration can induce other schizophrenia-like symptoms, namely positive, negative, and cognitive. Therefore, we set up to test whether repeated methionine administration results in behavioral responses that reflect schizophrenic-like symptoms. We show that methionine administration in adult mice induces behaviors that are related to the 3 types of schizophrenia symptoms and that these behavioral deficits can be reversed by antipsychotic administration. Our data support that repeated methionine administration may serve as a model for schizophrenia.

## Materials and Methods

### Animals

Male Swiss Webster mice, 8 to 11 weeks old, were obtained from Charles River Laboratories (Wilmington, MA). Mice were group-housed and maintained on a 12-hour-light/-dark cycle (light on at 7:00 am) with food and water available ad libitum. All experimental procedures were approved by the Institutional Animal Care and Use Committee of University of California, Irvine and were performed in compliance with national and institutional guidelines for the care and use of laboratory animals.

### Drug Administrations

MET (Sigma-Aldrich) was dissolved in saline. Haloperidol (HAL, Research Biochemicals International) and clozapine (CLZ, Sigma-Aldrich) were dissolved in saline with 0.3% tartaric acid, after which the pH was adjusted to pH 5–6 with sodium hydroxide. MET (750mg/kg, 15mL/kg, i.p.) was administered twice a day (9:00 am/3:00 pm) for 7 consecutive days. In the locomotion test, HAL (0.1, 0.25, 0.5mg/kg, 5mL/kg, i.p.) and CLZ (1, 2.5, 5mg/kg, 5mL/kg, i.p.) were administered 40 minutes before the test. In the forced swim test and PPI test, HAL (0.1, 0.25, 0.5mg/kg, 5mL/kg, i.p.) and CLZ (1, 2.5, 5mg/kg, 5mL/kg, i.p.) were administered 60 minutes before the test. In the novel objects recognition test, HAL (0.1, 0.25, 0.5mg/kg, 5mL/kg, i.p.) and CLZ (1, 2.5, 5mg/kg, 5mL/kg, i.p.) were administered 30 minutes before the training test.

### Behavioral Testing

#### Locomotion and Stereotypy Assays

The effect of MET on locomotor activity and stereotypy was assessed 18 hours after the last MET injection as described before (McNamara et al., 2006). Mice were placed into a locomotion test chamber (40×40cm, Med Associates, Inc.) and allowed to habituate for 30 minutes before test. For HAL and CLZ dose response experiments, the habituation step was skipped because of the known effect of both drugs decreasing spontaneous locomotor activity. The horizontal, vertical, and stereotypic activities for 1 hour were recorded and analyzed by Activity Monitor 5 software (Med Associates, Inc.).

#### Social Interaction Assay

The social interaction assay was carried out 18 hours after the last MET injection as described before (Kaidanovich-Beilin et al., 2011). The apparatus for the social interaction test is comprised of a rectangular 3-chamber Plexiglas box (manufactured by carpentry facility, University of California, Irvine). Each chamber is 20×40×20, cm and the dividing walls are made with a movable door in the middle with a 5-cm opening, which allows free access to each chamber. Two empty wire-mesh containment cups (9cm diameter × 10cm height) were placed in the middle of the right or left chamber (one per each side). The subject mice were first placed in the middle chamber and allowed to explore for 5 minutes with the dividing doors closed. A control mouse (an unfamiliar mouse of the same strain, gender, and age had no prior contact with the subject mouse) was placed inside the containment cup that is located in one of the side chambers. The placement of the control mouse in the side chambers was counter-balanced between trials. After habituation, the dividing doors were removed between the compartments to allow free access for the subject mouse to explore the 3 chambers for 10 minutes. The duration and number of direct contacts between the subject mice with both cups were recorded individually. Direct contact between the subject mouse and the cup or the body of the subject mouse except for the tails in an area 3cm around the cup was counted as an active contact. Tests were video recorded and analyzed by ANY-MAZE software (Stoelting Co.).

#### Forced Swim Assay

The forced swim assay was performed 18 hours after the last MET injection as previously described (Can et al., 2012). Mice were placed individually in a transparent glass cylinder containing water (24cm high, 14.5cm diameter, 14cm water depth) at 23°C to 25°C and forced to swim. Mice were videotaped for 6 minutes, and the immobility time (time spent passively floating) is recorded for the last 4 minutes, after discarding activity in the first 2 minutes during which an animal tries to escape. ANY-MAZE software was used to record and analyze immobility (Stoelting Co.).

#### PPI Assay

PPI test was measured 18 hours after the last MET injection as previously described (Duangdao et al., 2009). Detailed instrument introduction and procedure are described in supplementary Methods and Materials.

#### Novel Object Recognition Assay

The novel object recognition assay was carried out 18 hours after the last MET injection as described previously (Stefanko et al., 2009). This task consists of a training phase and a testing phase. Before training, all mice were handled 1 to 2min/d for 3 days and were habituated to the experimental apparatus 10min/d for 3 consecutive days without objects (2 hours after the morning MET injection). The experimental apparatus is a rectangular open field (20×40×20cm, manufactured by carpentry facility, University of California, Irvine). During the training phase, mice were placed in the experimental apparatus with 2 identical objects (PVC male pipe adapter, white, 1.5 inch×2.2 inch; PVC female hose mender, green, 1.4 inch×2.2 inch) and allowed to explore for 10 minutes. Exploration was defined as occurring when an animal faced an object by 1 inch or less or when any part of the animal body touched the object, except for the tail. The objects were thoroughly cleaned with 10% ethanol and then dried between trials to make sure no olfactory cues were present. Twenty-four hours later, mice were given the retention test. During these retention tests, mice were allowed to explore the experimental apparatus for 5 minutes in the presence of one familiar and one novel object. The location of the novel object was counterbalanced between trials. Duration and the number of times that the mice explored familiar or novel object were recorded individually. The relative exploration time was recorded and expressed by a discrimination index: (T_novel_−T_familiar_)/ (T_novel_ + T_familiar_)×100%. Tests were video recorded and analyzed by ANY-MAZE software (Stoelting Co.).

#### Inhibitory Avoidance Assay

Mice were tested for inhibitory avoidance 18 hours after the last MET injection as described previously (Chatterjee et al., 2011). The apparatus consisted of a chamber divided into 2 compartments, light illuminated and dark compartment with a guillotine door between the chambers (manufactured by carpentry facility, University of California, Irvine). The dark compartment has a stainless-steel floor through which a foot-shock can be delivered. In the training trial, mice were placed in the light compartment and the guillotine door was opened. After the mouse entered the dark chamber, the door was closed and foot shock (0.4 milliamps/1 s) was delivered. The animals remained in the dark compartment for 30 seconds. In the test trial, 48 hours after the training, mice were placed in the light compartment and their latency to enter the dark compartment (associated with the foot shock) was recorded as an index of memory consolidation. Cut-off latency was 10 minutes.

#### Open Field Assay

The open field assay was carried out 18 hours after the last MET injection as described before with slight modifications (Lipkind et al., 2004). Briefly, mice were placed into the open field test chamber (40×40cm, Med Associates, Inc.) and the total distance animals travelled for 10 minutes was recorded and analyzed by Activity Monitor 5 software (Med Associates, Inc.). The central zone was defined as a 24×24cm square in the middle of test chamber.

#### Rotarod Assay

The rotarod assay was carried out 18 hours after the last MET injection as described before with slight modifications (Duangdao, Clark et al., 2009). Mice were briefly trained to maintain their position on the rotarod apparatus (TSE Systems, Inc.) before the test session. The training session consisted of a 5-minute interval with an initial speed of 10 rounds/min (rpm) for 110 seconds. The rotarod then accelerated linearly from 10 to 20rpm in 80 seconds and the rod kept rotating with 20rpm for another 110 seconds. Sixty minutes after the training session, mice were placed on the rotarod at an initial speed of 4rpm. The test session consisted of a 7-minute interval, during which the rotarod accelerated linearly from 4 to 60rpm. The latency and rotation speed at which an animal fell off the rod was recorded automatically by an infrared beam located below the rotating rod.

#### Tail Flick Assay

The tail flick assay was carried out 18 hours after the last MET injection as described before (Zhang et al., 2014). In brief, acute nociceptive response was measured by using an electronically controlled tail-flick analgesimeter (UGO Basile Biological Research Apparatus, 7360 Tail Flick) that integrated both a thermal nociceptive stimulus and an automated response timer. A thermal stimulus (focused light from a 20W infrared bulb as the heat source) was applied to the tips of mice tails. The time from onset of stimulation to a rapid flick or withdrawal of the tail from the heat source was recorded as tail flick latency. A maximum of 22 seconds was set as a cut off time to prevent tissue damage to the animals.

### Data Analysis

Graphpad Prism (GraphPad Software, Inc.) was used for statistical analysis. Data are presented as means ± SEM. Results were analyzed by *Student t test* or ANOVA followed by the appropriate post hoc comparisons, and *P*<.05 was considered statistically significant.

## Results

### Behavioral Phenotype of MET-Injected Mice

MET (750mg/kg) or saline was administered to mice twice a day for 7 days. Mice were then observed and subjected to different behavioral assays to evaluate their phenotypes as they relate to psychotic syndromes.

**Figure 1.**
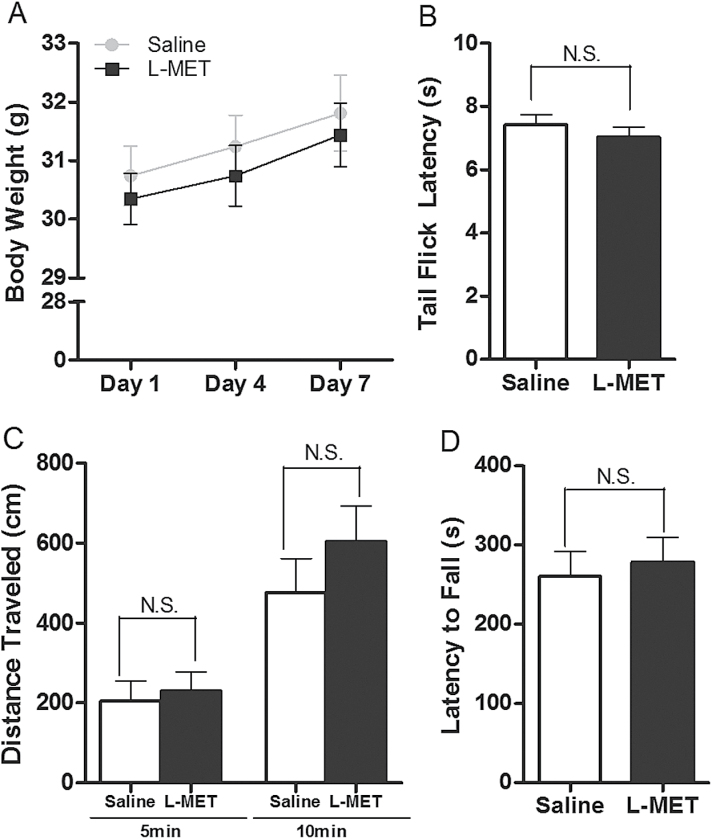
General effects of methionine (MET; 750mg/kg, i.p. twice per day, 7 days) administration. (A) Body weight during 7 days of methionine injections. Two-way ANOVA revealed no significant drug effects: F_1,36_ = 0.32, *P*=.5768. Data are presented as means±SEM (n=10). (B) Nociceptive response in tail flick test: t = 0.8488, *P*=.4072. Unpaired Student *t* test: saline vs MET, N.S., not significant. Data are presented as means±SEM (n=10). (C) Distance travelled in central area of the open field box in the first 5 and 10 minutes: 5 minutes, t = 0.3826, *P=*.7077; 10 minutes, t = 1.031, *P=*.3201. Unpaired Student *t* test: saline vs MET, N.S., not significant. Data are means±SEM (n=8). (D) Latency to fall in the rotarod test: t = 0.4135, *P=*.6855. Unpaired Student *t* test: saline vs MET, N.S. not significant. Data are presented as means±SEM (n=8).

The MET treatment did not have any evident pathological effects nor did it affect the body weights (*P*>.05; Figure 1A). When compared with control mice, MET-treated mice displayed the same level of nociceptive response in tail flick assay (*P*>.05; Figure 1B), spent the same amount of time tested in the center area in the open field assay (*P*>.05; Figure 1C) and the same level of motor coordination in the rotarod assay (*P*>.05; Figure 1D).

The MET treatment, however, did affect several behavioral responses that mimic schizophrenia symptoms.

#### Positive Symptoms

**Figure 2.**
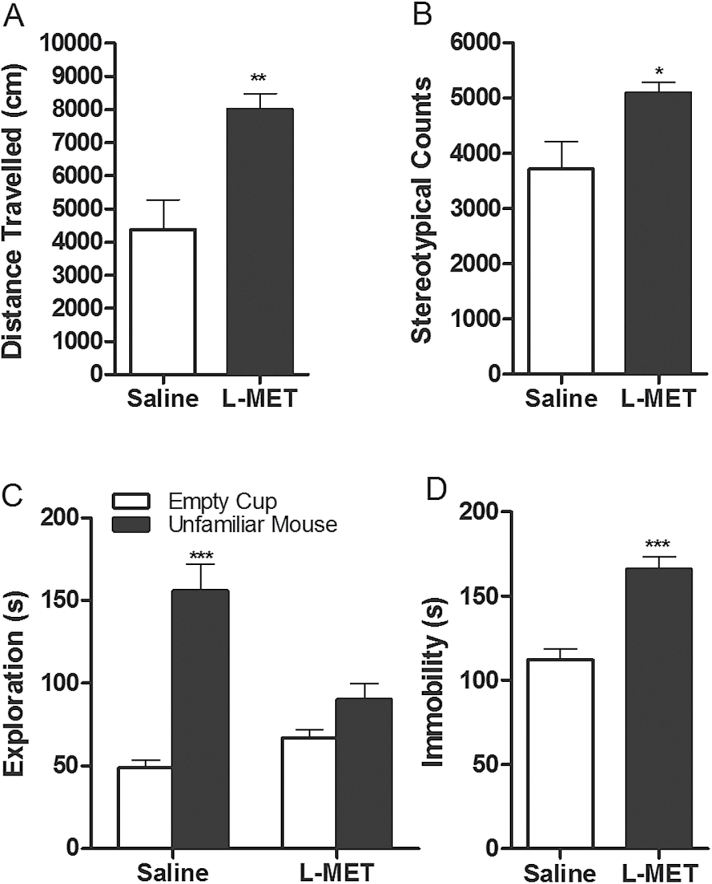
Effect of methionine (MET) on locomotion, stereotypy, social interaction and forced swim assays. (A) Distance mice travelled in 60 minutes of locomotion test: t=3.429, *P=*.0037. Unpaired Student *t* test: saline vs MET, **P<.01. Data are presented as means±SEM (n=8–9). (B) Stereotypic counts in 60 minutes of locomotion test: t = 2.450, *P=*.027. Unpaired Student *t* test: saline vs MET,*P<.05. Data are presented as means±SEM (n=8–9). (C) Time mice spent interacting with empty cup and control mice in social interaction test. Two-way ANOVA revealed significant drug effects: F_1,48_=5.58, *P=*.0222. Bonferroni posthoc test: empty cup vs control mice, ***P<.001. Data are presented as means±SEM (n=13). (D) Immobile time in forced swimming test: t = 5.264, ***P<.0001. Unpaired Student *t* test: saline vs MET, ***P<.001. Data are presented as means±SEM (n=9).

MET-treated mice exhibited a 2-fold increase in their locomotor activity, indicated by the increase in total distance that they travelled (*P*<.01; Figure 2A). They also displayed a greater level of stereotypic behaviors (*P*<.05; Figure 2B). These results suggest that MET-treated mice are hyperactive and display behavioral phenotypes that are related to the positive symptoms of schizophrenia.

#### Negative Symptoms

In the social interaction assay, the MET-treated mice displayed less interaction with the unfamiliar mice (*P*>.05; Figure 2C) than did control mice (*P*<.001; Figure 2C). These results indicate that the MET-treated mice exhibit impaired sociability. In the forced swim assay, MET-treated mice displayed more immobility time than control mice (*P*<.001; Figure 2D), suggesting a lower level of motivated affection. These assays reflect the negative symptoms of schizophrenia.

#### Sensorimotor Gating Behavior and Cognitive Symptoms

**Figure 3.**
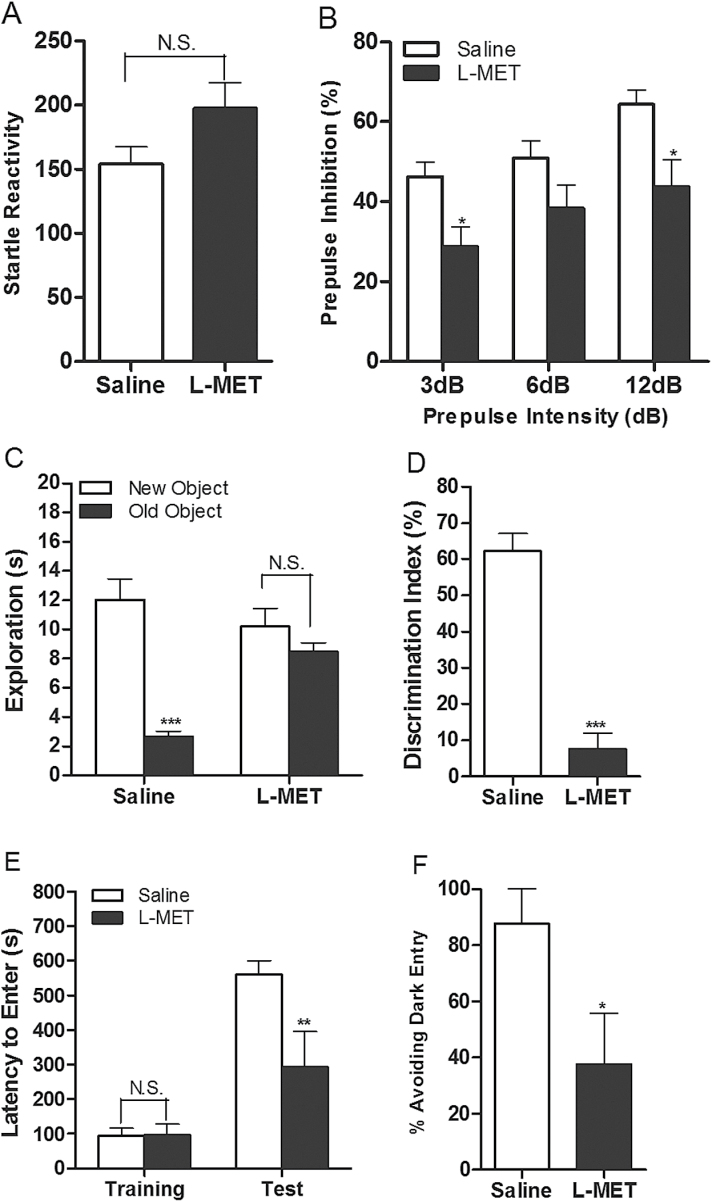
Effect of methionine (MET) on prepulse inhibition, novel object recognition, and inhibitory avoidance assays. (A) Startle reactivity: t = 1.795, *P=*.0781. Unpaired Student *t* test: saline vs MET, N.S., not significant. Data are presented as means±SEM (n=29). (B) Prepulse inhibition ratio. Two-way ANOVA revealed significant drug effects: F _1,162_=17.56, *P*<.0001. Bonferroni posthoc test: saline vs MET, *P<.05. Data are presented as means±SEM (n=28). (C) Time mice spent exploring both the new and old objects during test session. Two-way ANOVA revealed no significant drug effect: F _1,36_=3.95, *P=*.0544. Bonferroni posthoc test: new object vs old object, ***P<.001, N.S., not significant. Data are presented as means±SEM (n=10). (D) Discrimination index: t = 8.379, *P*<.0001. Unpaired Student *t* test: saline vs MET, ***P<.001. Data are presented as means±SEM (n=10). (E) Latency to enter the dark chamber in both training and retention session. Two-way ANOVA revealed significant drug effects: F _1,28_=5.38, *P=*.0279. Bonferroni posthoc test: saline vs MET, **P<.01, N.S., not significant. Data are presented as means±SEM (n=8). (F) Percentage of animals avoiding entering dark chamber in retention session: t = 2.256, *P=*.0406. Unpaired Student *t* test: saline vs MET, *P<.05. Data are presented as means±SEM (n=8).

We studied the effect of MET treatment on sensorimotor gating behavior. MET-treated mice did not display a significant change in their startle reactivity compared with control mice (*P*>.05; Figure 3A), yet they exhibited significantly decreased PPI ratios (*P*<.05; Figure 3B), suggesting an impairment in sensorimotor gating function. In the novel object recognition assay, the MET treatment did not affect the time mice spent exploring the identical objects during the training session (data not shown). However, during the retention session, when exposed to a new and an old object, the MET-treated mice did not discriminate between these 2 objects as did the control mice (*P*>.05; Figure 3C; *P*<.001; Figure 3D). These results indicate that MET-treated mice exhibit an impairment in recognition. In the inhibitory avoidance assay, saline- and MET-treated mice did not display differences in the latency to enter the dark chamber during the training trial. However, 48 hours after the training, MET-treated mice displayed a decrease in their latency time to enter the dark chamber (*P*<.01; Figure 3E) and a decreased percentage of animals avoiding entering the dark chamber (*P*<.05; Figure 3F) compared with control mice. Together, these last assays are indicative of cognitive deficits.

### Effect of Antipsychotics on the MET-Induced Schizophrenic-Like Phenotype in Mice

Because MET-treated mice displayed behaviors reflecting schizophrenic-like symptoms, we investigated the effects of 2 antipsychotic drugs on these behaviors. We chose HAL and CLZ as representative of the typical and atypical antipsychotics, respectively. Dose response experiments were first carried out on control mice for both drugs in each assay to determine the appropriate dose to administer (supplementary Figures S1–S4).

**Figure 4.**
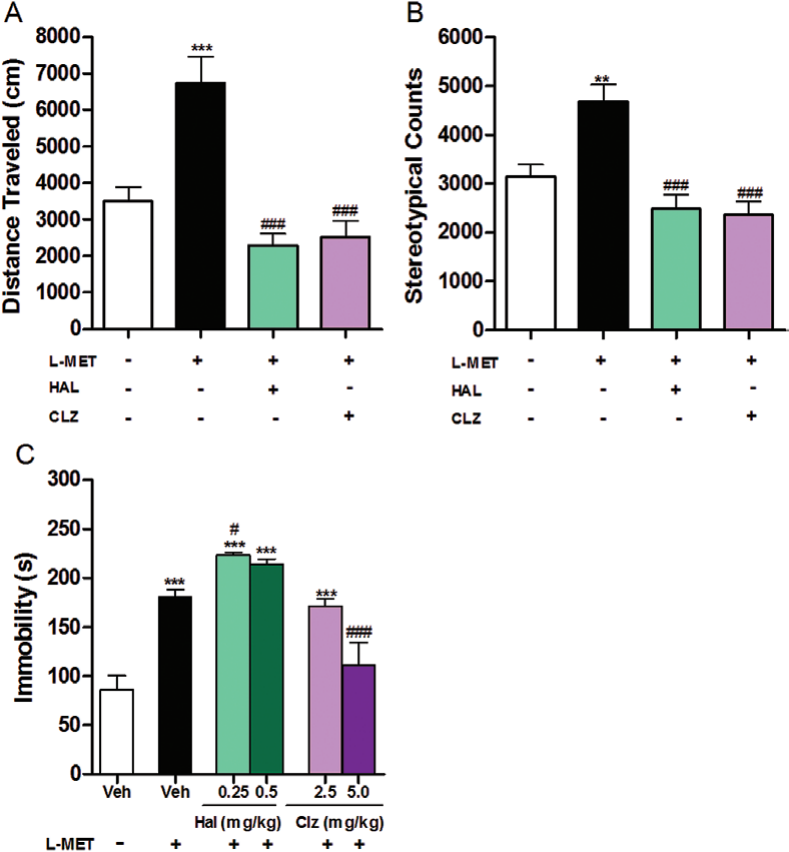
Effect of haloperidol (HAL) and clozapine (CLZ) on locomotion, stereotypy, and forced swim assays in methionine treated mice. (A) Effect of HAL (0.1mg/kg, i.p.) and CLZ (1.0mg/kg, i.p.) on distance mice travelled of locomotion test in methionine (MET)-treated mice. One-way ANOVA revealed a significant drug effect: F _3,33_=15.70, *P*<.0001. Bonferroni posthoc test: vehicle /- vs other 3 drug groups, ***P<.001; MET vs MET+HAL (CLZ), ^##^ *P*<.01, ^###^ *P*<.001. Data are presented as means±SEM (n=7–11). (B) Effect of HAL (0.1mg/kg, i.p.) and CLZ (1.0mg/kg, i.p.) on stereotypic counts of locomotion test in MET-treated mice. One-way ANOVA revealed a significant drug effect: F _3,33_=12.85, *P*<.0001. Bonferroni posthoc test: vehicle /- vs other 3 drug groups, **P<.01; MET vs MET+HAL (CLZ), ^###^ *P*<.001. Data are presented as means±SEM (n=7–11). (C) Effect of HAL (0.25, 0.5mg/kg, i.p.) and CLZ (2.5, 5.0mg/kg, i.p.) on immobile time of forced swimming test in MET-treated mice. One-way ANOVAs revealed significant drug effects: vehicle/- vs other 5 drug groups, F _5,53_=19.58, *P*<.0001. Dunnett’s posthoc test: ***P<.001; MET vs MET+HAL (CLZ), F_4,43_=12.86, *P*<.0001. Dunnett’s posthoc test: ^#^ *P*<.05, ^###^ *P*<.001. Data are presented as means±SEM (n=8–15).

HAL and CLZ at doses of 0.1mg/kg and 1mg/kg, respectively, were found to have no significant effect on the locomotion and stereotypy on naïve mice (*P*>.05; supplementary Figure S1) and were therefore selected. At these doses, a single injection of HAL or CLZ reversed the MET-induced locomotor hyperactivity and stereotypic behavior (*P*<.001; Figure 4A-B).

Neither HAL (0.1, 0.25, 0.5mg/kg) nor CLZ (1, 2.5, 5mg/kg) affected the immobility of naïve mice in the forced swim assay (*P>.*05; supplementary Figure S2). However, HAL at 0.25 (*P*<.05; Figure 4C) and 0.5mg/kg increased the immobility time of MET-treated mice. CLZ 2.5mg/kg did not affect the immobility time of MET-treated mice (*P*>.05; Figure 4C). In contrast, 5mg/kg CLZ decreased their immobility time (*P*<.001; Figure 4C).

**Figure 5.**
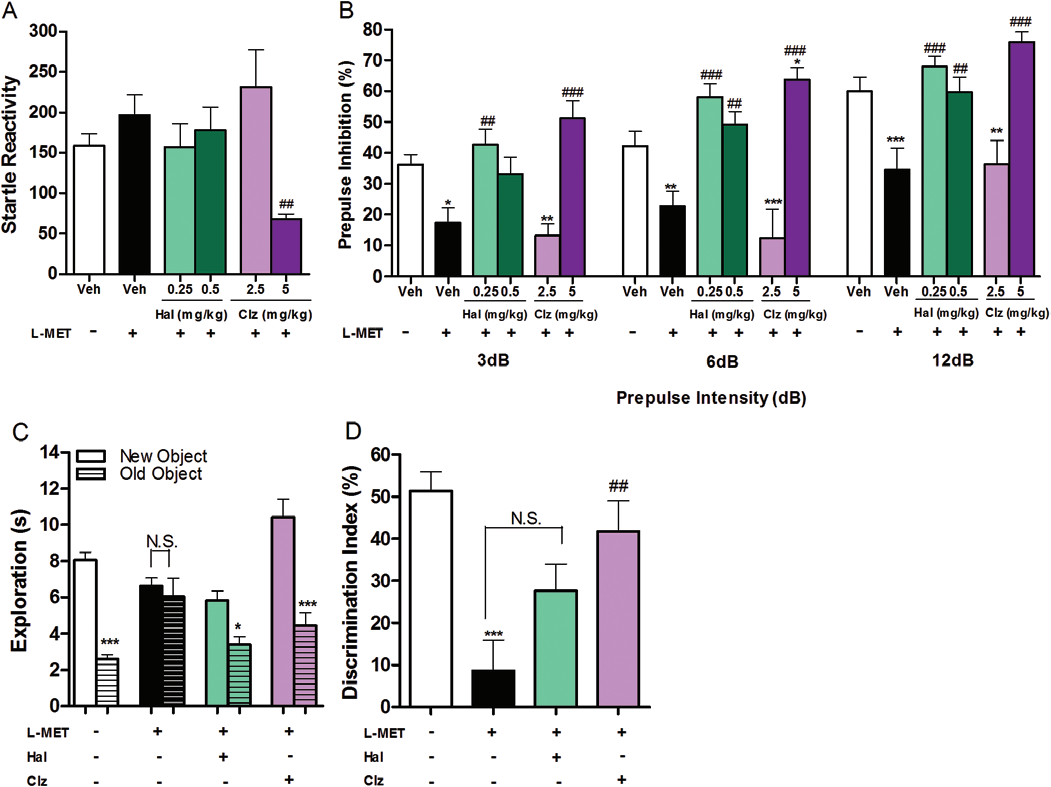
Effect of haloperidol (HAL) and clozapine (CLZ) on prepulse inhibition (PPI) and novel object recogniton assays in methionine (MET)-treated mice. (A) Effect of HAL (0.25, 0.5mg/kg, i.p.) and CLZ (2.5, 5.0mg/kg, i.p.) on startle reactivity in MET-treated mice. One-way ANOVAs revealed significant drug effects: vehicle/- vs other 5 drug groups, F_5,74_=3.768, *P=*.0043; MET vs MET+HAL (CLZ), F_4,57_=.016, *P=*.0061. Dunnett’s posthoc test: MET vs MET+HAL (CLZ), ^##^ *P*<.01. Data are presented as means±SEM (n=10–18). (B) Effect of HAL (0.25, 0.5mg/kg, i.p.) and CLZ (2.5, 5.0mg/kg, i.p.) on PPI ratio in MET-treated mice. Two-way ANOVAs revealed significant drug effects: vehicle/- vs other 5 drug groups, F_5,228_=29.01, *P*<.0001. Bonferroni posthoc test: *P<.05, **P<.01, ***P<.001; MET vs MET+HAL (CLZ), F_4,177_=33.50, *P*<.0001. Bonferroni posthoc test: ^##^ *P*<.01, ^###^ *P*<.001. Data are presented as means±SEM (n=11–19). (C) Effect of HAL (0.1mg/kg, i.p.) and CLZ (1.0mg/kg, i.p.) on time mice spent exploring both the new and old objects during test session in MET-treated mice. Two-way ANOVA revealed significant drug effects: F_3,56_=6.85, *P=*.0005. Bonferroni posthoc test: new object vs old object, *P<.05, ***P<.001, N.S., not significant. Data are presented as means±SEM (n=8). (D) Effect of HAL (0.1mg/kg, i.p.) and CLZ (1.0mg/kg, i.p.) on discrimination index in MET-treated mice. One-way ANOVA revealed a significant drug effect: F_3,28_=8.150, *P=*.0005. Bonferroni posthoc test: vehicle vs other 3 drug groups, ***P<.001; MET vs MET+HAL (CLZ), ^##^ *P*<.01, N.S., not significant. Data are presented as means±SEM (n=8).

In the PPI assay, we found that neither HAL (0.1, 0.25, 0.5mg/kg) nor CLZ (1, 2.5, 5mg/kg) affected the PPI ratio of naïve mice (*P*>.05; supplementary Figure S3B). HAL (0.25 and 0.5mg/kg) was able to reverse the PPI deficit induced by the MET treatment (*P*<.01; Figure 5B) as was CLZ 5mg/kg (*P*<.001; Figure 5B). At this dose CLZ, however, decreased the startle reactivity (*P*<.01; Figure 5A). CLZ 2.5mg/kg did not affect the PPI deficit induced by the MET treatment (*P*>.05; Figure 5B). In the novel object recognition assay, neither HAL 0.1mg/kg nor CLZ 1mg/kg affected the time naïve mice spent exploring identical or different objects or their discrimination index (*P*>.05; supplementary Figure S4). At these doses, only CLZ reversed the object recognition deficit found in MET-treated mice in terms of discrimination index P<.01; (Figure 5D).

## Discussion

The methionine cycle, as a primary modulator of methylation, may play a role in the etiology of schizophrenia. Associations between schizophrenia and genes encoding transmethylation enzymes have been reported (Roffman et al., 2008; Lajin et al., 2012; Saradalekshmi et al., 2014). Indeed in the sixties, the transmethylation hypothesis of schizophrenia had proposed that abnormal methylated metabolites of dopamine, noradrenaline, and serotonin might be responsible for the psychotic symptoms (Osmond and Smythies, 1952). Moreover, several studies have reported that methionine administration to schizophrenic patients exacerbates the psychotic symptoms (Pollin et al., 1961; Brune and Himwich, 1962; Spaide et al., 1969; Ananth et al., 1970; Antun et al., 1971; Cohen et al., 1974). Finally, more recently, it was shown that prolonged methionine treatment in mice produces behavioral responses that mimic certain aspects of schizophrenia (Tremolizzo et al., 2002). These data prompted us to study whether repeated methionine administration may result in a phenotype that reflects schizophrenia-like symptoms in mice.

We chose a methionine dose and treatment schedule that were used previously by Tremolizzo et al. (2002) that corresponded to the regimens previously administered to humans (Pollin et al., 1961; Brune and Himwich, 1962; Spaide et al., 1969; Ananth et al., 1970; Antun et al., 1971; Cohen et al., 1974). We show that this treatment has no effect on body weight, pain, or anxiety (Figure 1). This treatment, however, affected several behavioral responses.

First, the methionine-treated mice displayed increases in locomotor activity and in stereotypic behavior (Figure 2A-B). Locomotor hyperactivity and stereotypic behavior to a certain extent mimic the psychomotor disturbance in patients with schizophrenia and are used to evaluate the positive symptoms of schizophrenia (Kokkinidis and Anisman, 1980; Hoffman, 1992). Second, the methionine-treated mice exhibited impairment in social interaction and increase in helplessness (Figure 2C-D). Social withdrawal, affective flattening, and anhedonia are prominent among negative symptoms in schizophrenics. The social interaction assay, which is based on the fact that mice prefer to spend more time with other mice (Moy et al., 2004), and the forced swim assay, which monitors a lowered affective state (Porsolt et al., 1977), can thus serve to model negative symptoms. These results are consistent with the studies by Tremolizzo et al. (2005) and Matrisciano et al. (2011), who reported that MET treatment for 7 and 15 days impaired social interaction. Third, the methionine-treated mice exhibited less exploration of a novel object in the object recognition test (Figure 3C-D) showed deficits in inhibitory avoidance acquisition (Figure 3E-F) and the sensorimotor gating in the PPI (Figure 3A-B). The novel object recognition assay is based on the propensity of rodents to explore a novel object more than a familiar one. Impairments in recognition of a previously encountered object have been described in patients with schizophrenia and in pharmacological and genetic models of schizophrenia (Gabrovska et al., 2003; de Lima et al., 2005; Ibi et al., 2010). In the inhibitory avoidance assay, the animal is exposed to an aversive event and needs to learn to avoid future encounters. Sensorimotor gating deficit is a core feature of schizophrenia (Swerdlow et al., 2006) and reflects an inability to filter nonrelevant sensory information (Swerdlow et al., 2001). The PPI assay is commonly employed in rodent models of schizophrenia as a correlate for psychosis. These 3 assays were used to evaluate the cognitive deficits associated with schizophrenia.

From these results we can infer that repeated administration of methionine can induce behavioral deficits that recapitulate some of the positive, negative, and cognitive schizophrenia-like phenotypes. The methionine treatment can serve to establish a mouse model that reproduces some of the core symptoms of schizophrenia and as such presents face validity. However, a reliable animal model of schizophrenia must predict responsiveness to current available antipsychotic drugs (predictive validity).

We therefore tested whether the repeated methionine administration model possesses predictive validity by assessing the reversal of the behavioral abnormalities by 2 well-established prototypical antipsychotic drugs that are known to show efficacy in humans. HAL is a D2 antagonist and is known to possess good efficacy against positive symptoms but poor efficacy against negative symptoms. CLZ, an atypical antipsychotic, has a broader pharmacological profile and better efficacy against negative symptoms and cognitive deficits of schizophrenia.

The positive symptoms (hyperactivity and increased stereotypic behavior), induced by MET, were reversed by HAL and CLZ at doses that did not induce any behavioral changes in naïve mice (Figure 4A-B; supplementary Figure S1). On the other hand, CLZ but not HAL reversed the negative symptoms (increased immobility) induced by MET (Figure 4C). Further, both HAL and CLZ reversed MET-induced PPI deficits (Figure 5B). However, the dose of CLZ that reversed the PPI deficits also affected startle reactivity in both naïve and MET-treated animal (Figure 5A; supplementary Figure S3A). We also have shown that CLZ but not HAL can reverse the memory deficit in the novel object recognition assay (Figure 5D). These findings are of particular interest, since these 2 drugs show dissimilarity in managing human cognitive symptoms. This also shows that the pharmacological profiles of the 2 drugs differentially impact the assays.

Our results are in agreement with the majority of preclinical and clinical studies that have shown that positive symptoms of schizophrenia are reversed by both typical and atypical antipsychotics (Crespo-Facorro et al., 2006; Bradford et al., 2010) and that atypical but not typical antipsychotics can reverse negative symptoms and cognitive deficits (Noda and Nabeshima, 2000; Chindo et al., 2012). While the dopamine-related system hyperactivity is involved in the positive symptoms, the negative symptoms and cognitive deficits are believed to be associated with anatomical and functional abnormalities in the frontal cortex. Interestingly, Dong et al., (2008) found that fronto-cortical reelin and GAD67 promoter hypermethylation induced in mice by repeated MET administration can be reversed by the atypical antipsychotic CLZ but not by the typical antipsychotic HAL. The mechanism by which CLZ improved MET-induced negative symptoms might, thus, be through the reversal of fronto-cortical reelin and GAD67 promoter hypermethylation.

Our PPI assay results are interesting. In human as well as in certain animal models of schizophrenia, PPI deficits are reversed by atypical antipsychotics such as CLZ, risperidone, and olanzapine but not by typical antipsychotics (HAL) (Oranje et al., 2002; Kinkead et al., 2005). However, in other animal models, HAL is effective in improving the PPI deficit (Dirks et al., 2003; Onogi et al., 2010). This is also the case in our model where HAL and CLZ reverse the PPI deficit. Our finding showing that CLZ decreased the startle response is in agreement with other studies that reported a similar effect of high doses of CLZ (2.5, 5, 10mg/kg) irrespective of the mice strains (Olivier et al., 2001; Dirks et al., 2003). This decrease in the startle response was explained by the sedative effect of CLZ in these mice (Olivier et al., 2001; Ouagazzal et al., 2001; Dirks et al., 2003).

While the construct validity is beyond the scope of this paper, DNA hypermethylation of certain genes might be the mechanism responsible for our behavioral findings. Previous studies by the Guidotti group (Tremolizzo et al., 2002) showed that MET treatment of mice induced an increase of brain SAM and reduced reelin and GAD_67_ of the GABAergic interneurons in various cortical and hippocampal areas. In the MET-treated mice, the number of methylated CpG dinucleotides in the reelin promoter was shown to be inversely correlated with the reelin expression in the brain (Tremolizzo et al., 2002). Tueting et al. (2010) showed that MET treatment of mice caused a decrease in the density of dendritic spine in layer III pyramidal neurons in the frontal cortex, which resembles the downregulation in spine density observed in the postmortem brains of patients with schizophrenia and in heterozygous reeler mice. Further studies are required to fully substantiate the construct validity of this model with regard to the involvement of different neurotransmitters’ systems, and neuronal functional changes.

In conclusion, we were able to show that the behavioral impairments induced by the repeated MET treatment are consistent with those observed in schizophrenia patients. We therefore propose MET treatment as an animal model recapitulating several symptoms of schizophrenia. Our model relies on the use of an essential amino acid that we consume daily and on an intervention that is relatively simple and time effective. We have established the face and predictive validity for this model, and it may offer an additional tool for assessing novel antipsychotics.

## Statement of Interest

None.

## Supplementary Material

supplementary Methods and Materials
