# Supplementary material for: A Methionine-Induced Animal Model of Schizophrenia: Face and Predictive Validity
Source: Int J Neuropsychopharmacol. 2015 May 19;18(12):pyv054. doi: 10.1093/ijnp/pyv054 (PMC4675974; doi:10.1093/ijnp/pyv054)
Supplement: supplementary Methods and Materials [file ijnp_pyv054_index.html]

Supplementary Data | International Journal of Neuropsychopharmacology

## Supplementary Data

Data files

- Supplementary Data - Supplementary Data
