# Supplementary material for: A Methionine-Induced Animal Model of Schizophrenia: Face and Predictive Validity
Source: Int J Neuropsychopharmacol. 2015 May 19;18(12):pyv054. doi: 10.1093/ijnp/pyv054 (PMC4675974; doi:10.1093/ijnp/pyv054)
Supplement: supplementary Methods and Materials [file pyv054Supplementary.doc]

**Supplementary Information**

**A Methionine-Induced Animal Model of Schizophrenia: Face and Predictive Validity**

Wang Lien, Alachkar Amal, Sanathara Nayna, Belluzzi James.D, Wang Zhiwei and Civelli Olivier

**SUPPLEMENTARY METHODS AND MATERIALS**

**Prepulse Inhibition Assay**

The startle chamber consists of a nonrestrictive Plexiglas cylinder resting on a platform inside of a ventilated and sound attenuated box. A high-frequency loudspeaker inside each chamber produced background noise of 65 dB as well as the various acoustic stimuli. Vibrations of the Plexiglas cylinder caused by the body startle response of the animal are converted into analog signals by a piezoelectric accelerometer attached to the platform. A total of 65 readings are recorded at 1-millisecond intervals beginning at the stimulus onset. Average amplitude over this time is used as the measure of startle. Calibration was performed before every use to ensure the accuracy of the sound levels and startle measurements.

During the test, mice were placed in the startle chambers for 5 minutes of acclimation with 65 dB background noise. The PPI session consisted of 5 different trials: no-stimulus trials, 3 prepulse trials, and startle trials. No-stimulus trials consist of background noise only (65 dB). Startle trials consist of a 40-millisecond duration startle stimulus at 120 dB (p120). Prepulse trials consist of a 20-millisecond duration prepulse at 68 dB (pp3), 71 dB (pp6), or 77 dB (pp12), a 100-millisecond interstimulus interval, followed by a 40-millisecond duration startle stimulus at 120 dB. Test sessions began with 5 presentations of the p120 trial, followed by 10 presentations of the no-stimulus trial, p120 trials, pp3, pp6, and pp12 prepulse trials given in a pseudorandom order with an intertrial interval of 8 to 23 seconds (mean 15 seconds) and ending with 5 presentations of the p120 trial. The amount of PPI is calculated as a percentage score for each acoustic prepulse intensity: % PPI = 100-([(startle response for prepulse + pulse trials)/ (startle response for pulse-alone trials)] × 100). The magnitude of the response was calculated as the average response to all of the startle or prepulse trials.

**SUPPLEMENTARY RESULTS**


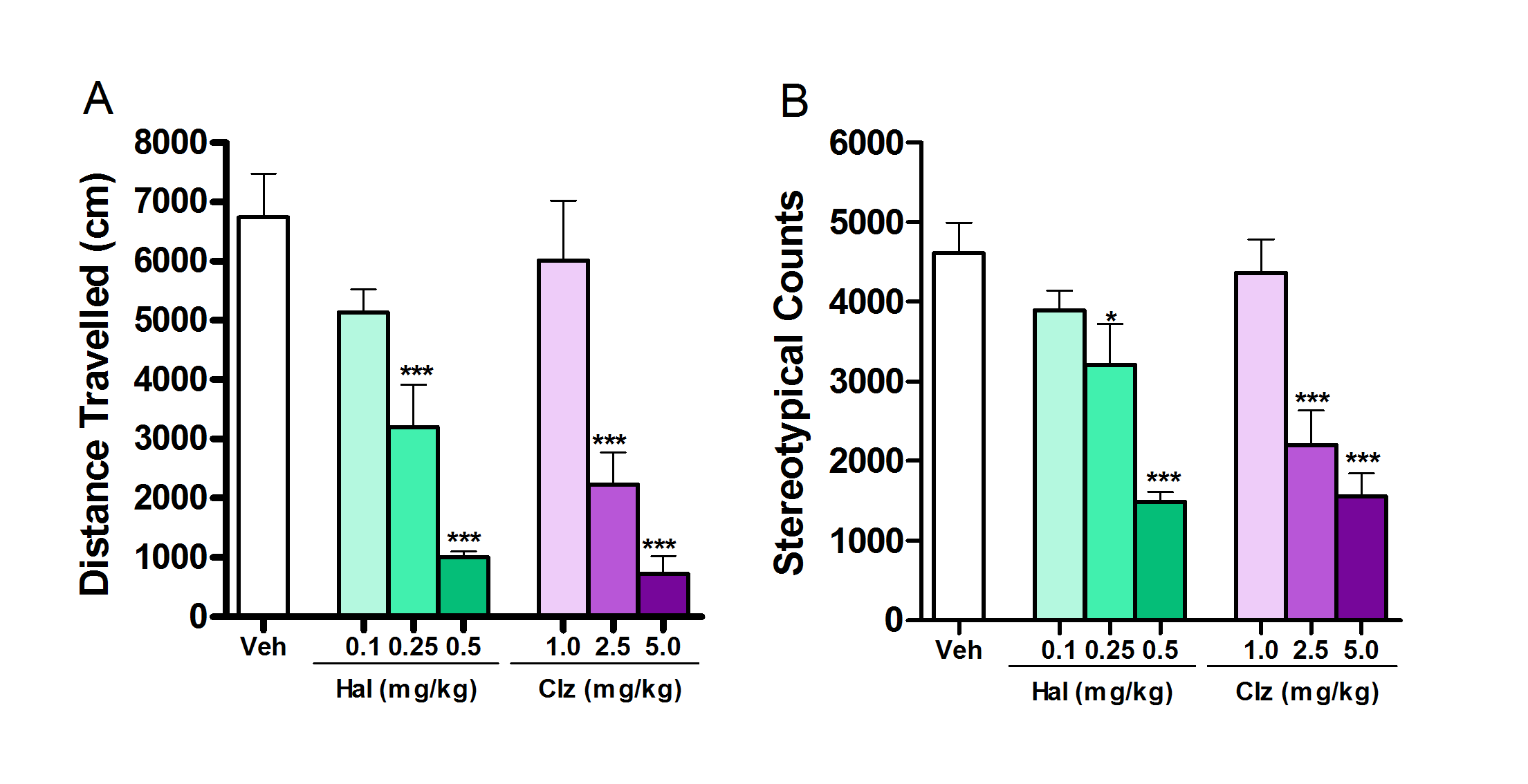
**Fig.S1**

Figure S1. Effect of haloperidol (HAL) and clozapine (CLZ) in locomotion and stereotypy assays in naïve mice. (A) Dose response of HAL (0.1, 0.25, 0.5 mg/kg, i.p.) and CLZ (1.0, 2.5, 5.0 mg/kg, i.p.) in distance mice travelled of locomotion test. One-way ANOVA revealed a significant drug effect: F_6,46_ = 12.93, *P* < .0001. Dunnett’s posthoc test: vehicle vs drug, *** *P* < .001. Data are presented as means ± SEM (n = 6-11). (B) Dose response of HAL (0.1, 0.25, 0.5 mg/kg, i.p.) and CLZ (1.0, 2.5, 5.0 mg/kg, i.p.) in stereotypic counts of locomotion test. One-way ANOVA revealed a significant drug effect: F_6,47_ = 10.31, *P* < .0001. Dunnett’s posthoc test: vehicle vs drug, * *P* < .05, *** *P* < .001. Data are presented as means ± SEM (n = 6-11).

**Fig.S2**

**
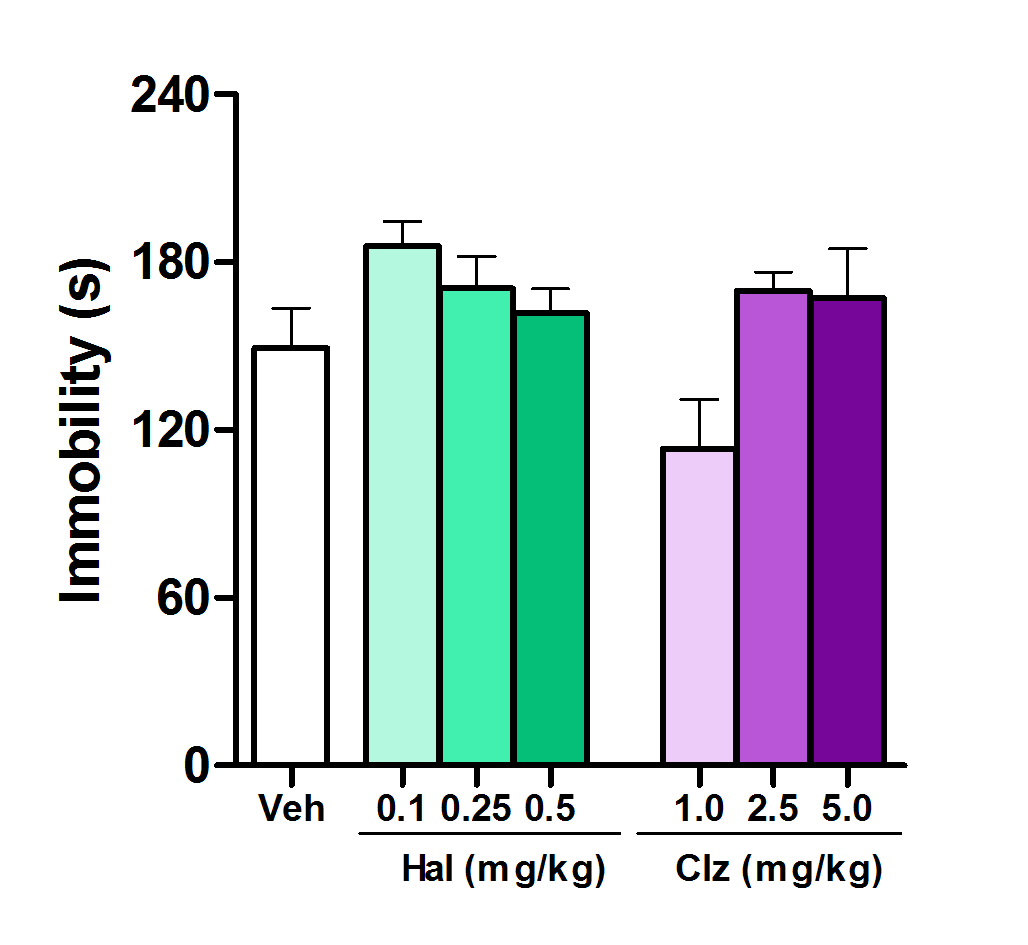
**

Figure S2. Effect of haloperidol (HAL) and clozapine (CLZ) on forced swim assay in naïve mice. Dose response of HAL (0.1, 0.25, 0.5 mg/kg, i.p.) and CLZ (1.0, 2.5, 5.0 mg/kg, i.p.) on forced swimming test in naïve mice. One-way ANOVA revealed a significant drug effect: F_6,51_ = 2.940, *P* = .0153. Dunnett’s posthoc test. Data are presented as means ± SEM (n = 7-10).


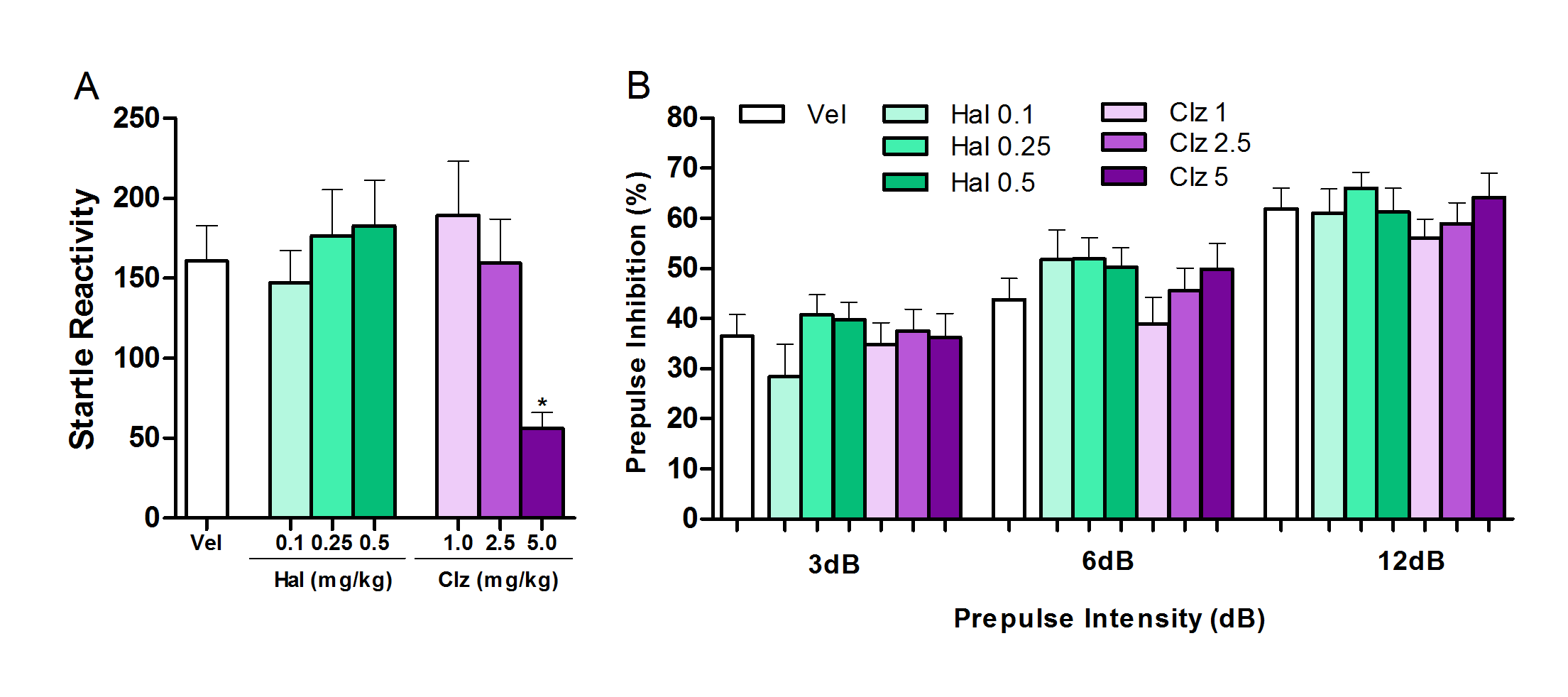
**Fig.S3**

Figure S3. Effect of haloperidol (HAL) and clozapine (CLZ) on prepulse inhibition test in naïve mice. (A) Dose response of HAL (0.1, 0.25, 0.5 mg/kg, i.p.) and CLZ (1.0, 2.5, 5.0 mg/kg, i.p.) in startle reactivity. One-way ANOVA revealed a significant drug effect: F_6,87_ = 2.532, *P* = .0263. Dunnett’s posthoc test: vehicle vs drug, **P* < .05. Data are means ± SEM (n = 11-17). (B) Dose response of HAL (0.1, 0.25, 0.5 mg/kg, i.p.) and CLZ (1.0, 2.5, 5.0 mg/kg, i.p.) in prepulse inhibition ratio. Two-way ANOVA revealed no significant drug effect: F_6,255_ = 1.43, *P* = 0.2029. Data are presented as means ± SEM (n = 10-16).

**Fig.S**
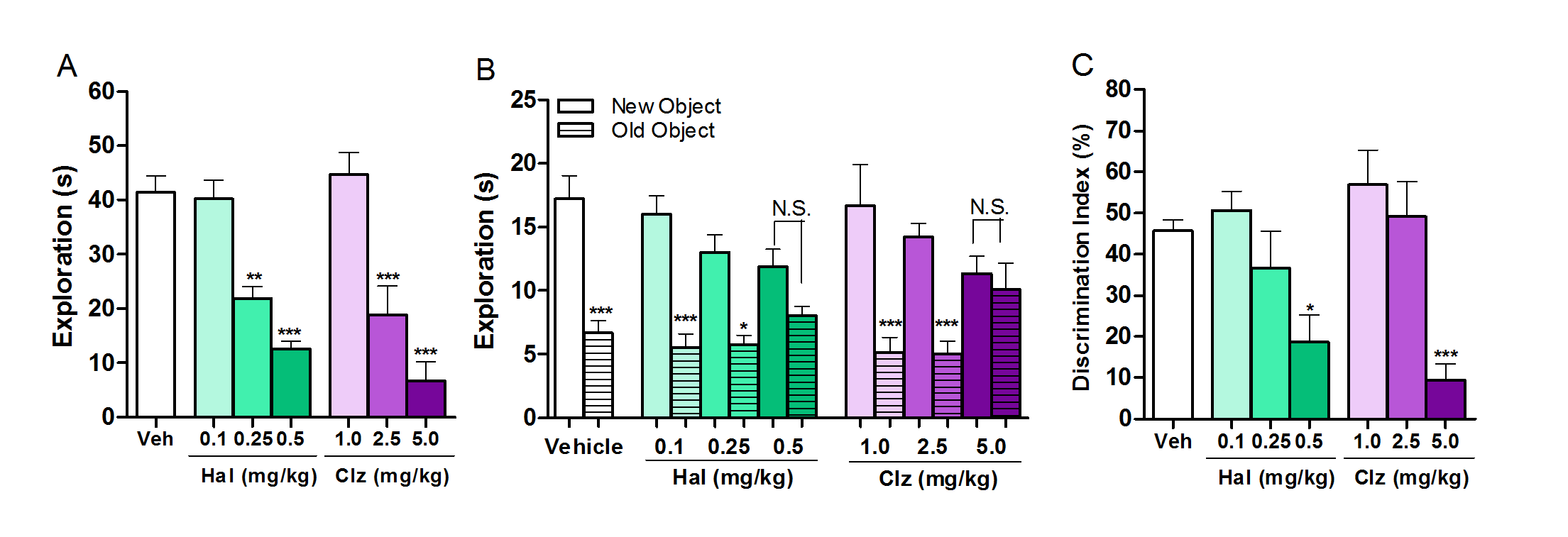
**4**

Figure S4. Effect of haloperidol (HAL) and clozapine (CLZ) on novel object recognition assay in naïve mice. (A) Dose response of HAL (0.1, 0.25, 0.5 mg/kg, i.p.) and CLZ (1.0, 2.5, 5.0 mg/kg, i.p.) in total exploration time on identical objects in training session. One-way ANOVA revealed a significant drug effect: F_6,45_ = 17.76, *P* < .0001. Dunnett’s posthoc test: vehicle vs drug, ***P* < .01, ****P* < .001. Data are presented as means ± SEM (n = 6-9). (B) Dose response of HAL (0.1, 0.25, 0.5 mg/kg, i.p.) and CLZ (1.0, 2.5, 5.0 mg/kg, i.p.) in time mice spent exploring both the new and old objects during test session. Two-way ANOVA revealed no significant drug effect: F_6,90_ = 0.72, *P* = .6343. Bonferroni posthoc test: new object vs old object, **P* < .05, ****P* < .001, N.S., not significant. Data are presented as means ± SEM (n = 6-9). (C) Dose response of HAL (0.1, 0.25, 0.5 mg/kg, i.p.) and CLZ (1.0, 2.5, 5.0 mg/kg, i.p.) in discrimination index. One-way ANOVA revealed a significant drug effect: F_6,45_ = 7.108, *P* < .0001. Dunnett’s posthoc test: vehicle vs drug, **P* < .05, ****P* < .001. Data are presented as means ± SEM (n = 6-9).
